# Review of the Efficacy and Mechanisms of Traditional Chinese Medicines as a Therapeutic Option for Ionizing Radiation Induced Damage

**DOI:** 10.3389/fphar.2021.617559

**Published:** 2021-02-15

**Authors:** Xiaomeng Zhang, Xiaoying Chen, Lei Wang, Changhao He, Zhongyu Shi, Qian Fu, Wenhui Xu, Shujing Zhang, Sumin Hu

**Affiliations:** ^1^ School of Traditional Chinese Medicine, Beijing University of Chinese Medicine, Beijing, China; ^2^ Beijing Academy of Traditional Chinese Medicine, Beijing University of Chinese Medicine, Beijing, China

**Keywords:** ionizing radiation, oxidative damage, Chinese herbal medicines, Chinese herbal prescriptions, anti-radiation, radiation protective drugs

## Abstract

Ionizing radiation damage refers to acute, delayed, or chronic tissue damage associated with ionizing radiation. Specific or effective therapeutic options for systemic injuries induced by ionizing radiation have not been developed. Studies have shown that Chinese herbal Medicine or Chinese Herbal Prescription exhibit preventive properties against radiation damage. These medicines inhibit tissue injuries and promote repair with very minimal side effects. This study reviews traditional Chinese herbal medicines and prescriptions with radiation protective effects as well as their mechanisms of action. The information obtained will guide the development of alternative radioprotectants.

## Introduction

**FIGURE 1:**
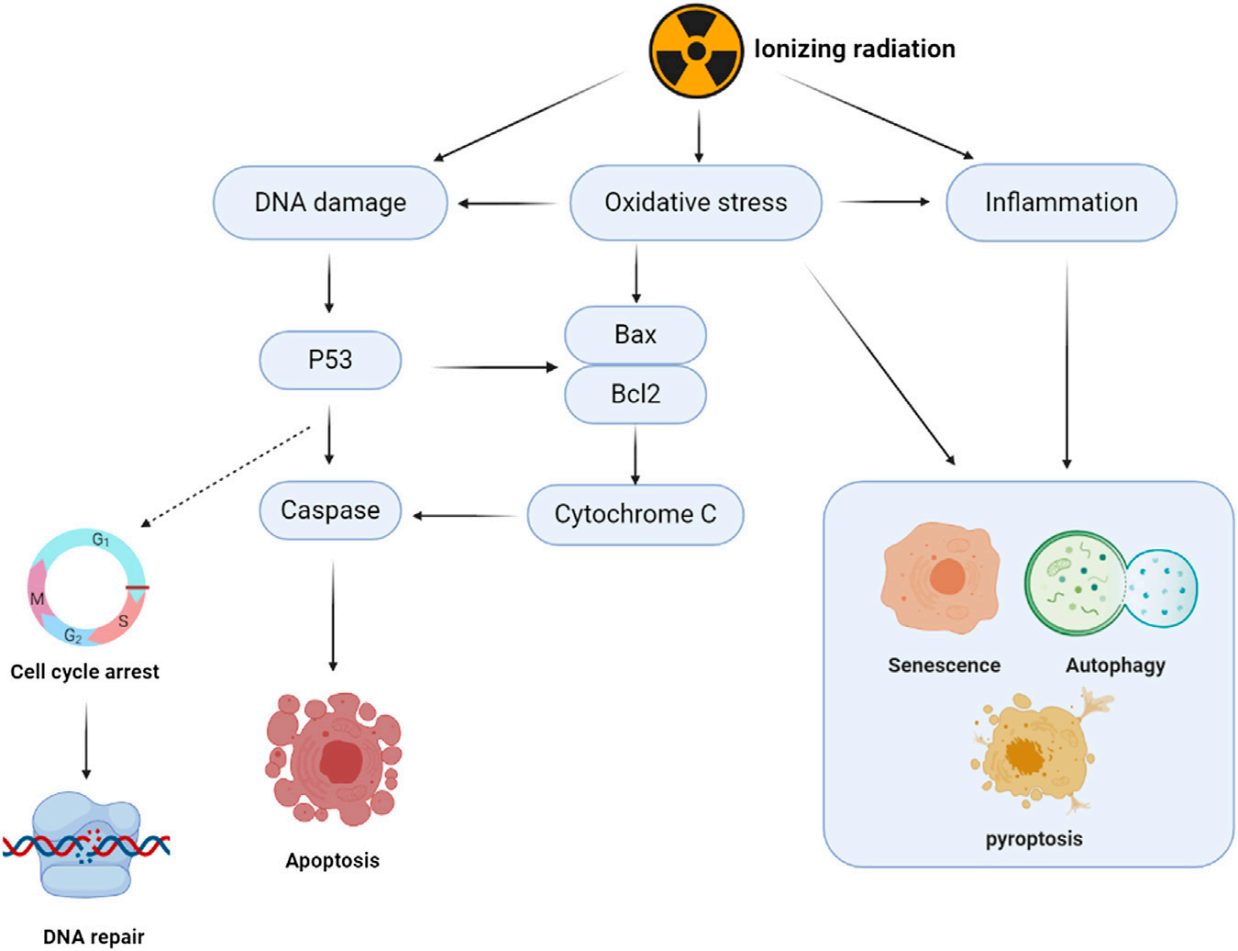
Schematic diagram of ionizing radiation damage mechanism. The damage of ionizing radiation to cell structure mainly has two ways: the direct damage to DNA and the indirect damage from accumulated ROS. DNA damage response and repair induced by ionizing radiation can lead to cell cycle arrest and activate DNA homologous recombination (HR) or non-homologous end joining (NHEJ) repair. DNA damage induces the phosphorylation of P53, promotes the expression of Bax and other apoptotic proteins, promotes the release of Cytochrome C from mitochondria, and activates Caspase from the intrinsic apoptosis pathway. P53 can induce the expression of downstream death receptors and death ligands, which triggers the extrinsic apoptosis pathway. Oxidative stress caused by ROS overproduction triggers DNA double-strand breaks that continue and exacerbate DNA damage. It also induces mitochondrial dysfunction and intrinsic apoptotic cascade reaction. Ionizing radiation increases the secretion of inflammatory factors and induces immune responses. Inflammatory factors and oxidative damage are involved in regulating cell fate, including autophagy, pyroptosis and senescence.

Ionizing radiation is essential in clinical diagnoses and treatment. It is an effective therapeutic strategy for cancer treatment. Approximately 50% of cancer patients are administered with radiotherapy to inhibit metastasis (Begg et al., 2011). Ionizing radiation causes clustered DNA damage and leads to persistent oxidative stress injuries to cellular macromolecules (Shuryak, 2019). Radiotherapy inhibits metastasis by inducing DNA damage. However, it causes unintended damage to normal cells by enhancing DNA double-strand breaks (DSBs). Double-strand breaks can be repaired through two major pathways: the non-homologous end-joining (NHEJ) and homologous recombination (HR). The NHEJ pathway occurs during the G0/G1 phase, while the HR repair pathway is only active in the late S and G2 phases (Shibata, 2017). Severe genetic changes such as chromosomal deletions and translocations in the repair process can stimulate tumorigenesis (Shrivastav et al., 2008; Gerelchuluun et al., 2015). According to the United Nations Scientific Committee, the effects of atomic radiation include mutations due to DNA deletions (Sankaranarayanan and Wassom, 2005), and epigenetic transmissions that affect generations (Horemans et al., 2019). Oxidative damage after exposure to ionizing radiation is a crucial reason for sustained injuries (Einor et al., 2016). Approximately 70% of cellular radiation damage is indirectly caused by water dissociation and reactive oxygen species (ROS). Free radicals are triggers for a state of constant oxidative stress (Anuranjani and Bala, 2014). Inflammation, immunity, and other associated signaling pathways are involved in the regulation of cellular damage by inducing cell senescence, apoptosis, and other cell fates (Santivasi and Xia, 2014). The pathological process is as shown in Figure 1. Ferroptosis is a novel form of cell death that is involved in pathological damage after irradiation (Lei et al., 2020). There is a need for the development of drugs that inhibit or repair pathological damages associated with radiation.

Current therapeutic strategies for radiation damage focus on minimizing DNA breaks, generation of antioxidants, scavenging free radicals, and inhibiting lipid peroxidation (Weiss and Landauer, 2003; Johnke et al., 2014; Smith et al., 2017). However, clinical applications of radioprotectants are limited because of its limited efficacies and severe side effects (Brizel, 2007; Citrin et al., 2010; Singh and Seed, 2019). Chinese herbal medicines have been used for thousands of years. They are widely used in clinical settings to treat various diseases, or are used in combination with western medicines to improve clinical efficacies (Li and Xu, 2011; Hao et al., 2017; Takayama and Iwasaki, 2017; Wang and Zhang, 2017; Yeh et al., 2017; Lei et al., 2019). As early as the 1960s, Chinese researchers had extended the research focused on the anti-radiation effects of Chinese herbal medicines. It has been found that *Panax ginseng C.A.Mey.*, *Ganoderma Lucidum Karst*, *Angelica sinensis (Oliv.) Diels*, and other medicinal herbs exhibit varying degrees of anti-radiation effects. In addition, Chinese Herbal Prescriptions have been shown to facilitate physical recovery. Traditional Chinese medicinal herbs have complex chemical structures and biological activities. The Chinese Herbal Prescription, which is composed of more than one medicinal herb, is the embodiment of traditional Chinese medicine’s clinical applications. The Chinese Herbal Prescriptions can enhance the efficacy of a drug, reduce or neutralize the adverse effects of individual drugs and improve their therapeutic efficacies (Shen et al., 2017; Li et al., 2019b; Shi et al., 2019b). In recent years, researches on the treatment of ionizing radiation damage with Traditional Chinese Medicine are regularly emerging. The active compounds in Chinese medicinal herbs and Chinese Herbal Prescriptions exhibit significant effects on the reduction of oxidative stress and promote DNA repair. Their biological mechanisms involve the regulation of multiple signaling pathways.

In this review, we summarized recent advances that have been aimed at elucidating the functions and mechanisms of effective active ingredients of Chinese herbal medicines and prescriptions in preventing ionizing radiation associated damage. We aimed at establishing novel therapeutic avenues for the development and clinical applications of radiation protective drugs.

## Anti-Radiation Active Compounds of Chinese Herbal Medicines

Active compounds in Chinese herbal medicines can scavenge for free radicals, reduce DNA damage, promote post-injury repair, and reduce cell apoptosis. Therefore, these herbs prevent radiation damage through different mechanisms. Below, we highlight a few representative active compounds and highlight their potential mechanisms and pharmacological activities in radioprotection (As shown in Table 1).

**TABLE 1:** Effects and Mechanisms of active compounds of Chinese Herbal Medicines in ionizing radiation damage.

Names	Category	Origins	Objects (model inducer,dose)	Pharmacological action/Mechanisms	References
Ginsenoside Rb(1), Rb(2), Rc, Rd, Re and Rg(1)	Saponins	*Panax ginseng C.A.Mey.*	ICR mice (12 Gy, 6.5 Gy ^60^Co γ rays)	Reduce intestinal crypt cells apoptosis	Lee et al. (2006)
Gypenosides	Saponins	*Gynostemma pentaphyllum*	Male Balb/c mice (5 Gy ^60^Co γ rays)	Increase the activity of SOD and CAT in serum, interference with Nrf2 signaling pathways	Ying et al. (2018)
Eleutheroside	Saponins	*Eleutherococcus senticosus (Rupr. & Maxim.) Maxim.*	Male mice (2 Gy x rays)	Improve peripheral blood cell radiation injury	Yue et al. (2005)
Acidic polysaccharide of ginseng	Polysaccharides	*Panax ginseng C.A.Mey.*	Bms (1 Gy ^60^Co γ rays) Balb/c mice (5 Gy ^60^Co γ rays)	Alter the phenotype of bms, increase the viability and alloreactivity of bms	Kim et al. (2007)
Acidic polysaccharide of ginseng	Polysaccharides	*Panax ginseng C.A.Mey.*	C57BL/6 mice(7 Gy ^60^Co γ rays)	Inhibit p53 dependent pathway and mitochondrial apoptosis pathway activation	Park et al. (2011) and Bing et al. (2014)
Lycium Barbarum Polysaccharide	Polysaccharides	*Lycium barbarum L.*	Wistar rats (2.3 Gy ^60^Co γ rays)	Elevate SOD levels, suppress MDA levels and restores testosterone levels	Luo et al. (2011)
Lycium Barbarum Polysaccharide	Polysaccharides	*Lycium barbarum L.*	Kunming mice (4 Gy X-rays)	Improve antioxidant capacity and cell cycle	Zhou et al. (2016)
Lycium Barbarum Polysaccharide	Polysaccharides	*Lycium barbarum L.*	Wistar rats (2.3 Gy ^60^Co γ rays)	Inhibit mitochondrial apoptosis	Luo et al. (2014)
Astragalus Polysaccharide	Polysaccharides	*Astragalus mongholicus Bunge*	Balb/c mice (5 Gy ^60^Co γ rays)	Inhibit the secretion of pro-inflammatory factors, reduce the damage of pulmonary fibrosis caused by peroxide	Liu et al. (2014)
Astragalus Polysaccharide	Polysaccharides	*Astragalus mongholicus Bunge*	Bmscs A549 cells (2 Gy X-rays)	Improve the ROS - mediated side effects of ionizing radiation via MAPK/NF-κB signaling pathway	Zhang et al. (2018c)
Astragalus Polysaccharide	Polysaccharides	*Astragalus mongholicus Bunge*	Human bone marrow mesenchymal stem cells (2 Gy X-rays)	Promote the self-renewal and proliferation of cells	Kongxi et al. (2020)
Astragalus Polysaccharide	Polysaccharides	*Astragalus mongholicus Bunge*	Human bone marrow mesenchymal stem cells (2 Gy ^12^C^6+^)	Down-regulates NF-κB signaling pathway to maintain cell DNA stability	Li-ying et al. (2018)
Ganoderma Polysaccharides	Polysaccharides	*Ganoderma Lucidum Karst*	Mice (5 Gy X-rays)	Improves the expression of biomarkers in the thymus	Kunmu et al. (2020)
Ganoderma Polysaccharides	Polysaccharides	*Ganoderma Lucidum Karst*	Kunming mice (7.5 Gy ^60^Co γ rays)	Increase the survival rate, improve the phagocytosis of mononuclear macrophages and NK cell activity	Guohui et al. (2004)
Angelica sinensis polysaccharide	Polysaccharides	*Angelica sinensis (Oliv.) Diels*	Balb/c male mice (3 Gy ^60^Co γ rays)	Increase the number of bone marrow cell and protect hematopoietic system	Zhao et al. (2012)
Gingko flavonoids	Flavonoid	*Ginkgo biloba L.*	SD rats (800 cgy X-rays)	Inhibit lipid peroxidation and reduce the secretion of LDH and TNF-α	Sener et al. (2006)
Houttuynia Cordata	Flavonoid	*Houttuynia cordata Thunb.*	Kunming mice (3 Gy ^60^Co γ rays)	Improve peripheral blood cell injury	Jun and Zhenghai (2010)
Paeoniflorin	Glycosides	*Paeonia lactiflora Pall.*	EA.hy926 cell line (10 Gy ^60^Co γ rays)	Interfere with Nrf2/HO-1 pathway to reduce oxidative stress response	Yu et al. (2013)
Paeoniflorin	Glycosides	*Paeonia lactiflora Pall.*	Thymocytes (0–8 Gy ^60^Co γ rays)	Act on the expression of Bcl-2, Bax and Caspase-3, and reduce mitochondrial apoptosis	Li et al. (2007)
Paeoniflorin	Glycosides	*Paeonia lactiflora Pall.*	HSF cell line (16 Gy X-rays)	Inhibits MAPK signal pathway to revers DNA damage	Yang et al. (2015)
Astragaloside IV	Glycosides	*Astragalus mongholicus Bunge*	Balb/c mice (6 Gy ^60^Co γ rays)	Regulates cell apoptosis or cell cycle, down-regulates Bax/Bcl-2 ratio, and reduces cell cycle arrest in G0/G1 phase	Li et al. (2011)
Rhodioloside	Glycosides	*Rhodiola rosea L.*	AHH-1 cells (4, 6, 8, 10 Gy ^60^Co γ rays)	Increases the proliferative activity of lymphocytes	Tianxiang et al. (2013)
Ferulic Acid	Phenolic acids	*Angelica sinensis (Oliv.) Diels*	Swiss albino mice (10 Gy ^60^Co γ rays)	Promotes Nrf2 nuclear translocation and activates the NHEJ repair pathway	Das et al. (2017a)
Ferulic Acid	Phenolic acids	*Angelica sinensis (Oliv.) Diels*	Swiss mice (4, 6, 8, 10 Gy ^60^Co γ rays)	Reduces DNA strand break in leukocytes and bone marrow cells and promotes the recovery of bone marrow hematopoietic functions	Maurya and Devasagayam (2013)
Ferulic Acid	Phenolic acids	*Angelica sinensis (Oliv.) Diels*	Swiss albino mice (10 Gy ^60^Co γ rays)	Reduces IκBα phosphorylation, and NF-κB nuclear translocation, improves radiation-induced inflammation	Das et al. (2014)
Ferulic Acid	Phenolic acids	*Angelica sinensis (Oliv.) Diels*	Swiss albino mice (10 Gy ^60^Co γ rays)	Reduces lipid peroxidation and increases the activity of SOD and catalase, increases PI3K phosphorylation levels, reduces cell cycle arrest	Das et al. (2016)
Ferulic Acid	Phenolic acids	*Angelica sinensis (Oliv.) Diels*	Swiss albino mice (2.5, 5, 10 Gy ^60^Co γ rays)	Inhibits peroxide and downstream mitochondrial apoptosis pathway activation	Das et al. (2017b)
Ferulic Acid	Phenolic acids	*Angelica sinensis (Oliv.) Diels*	SD rats (5 Gy ^60^Co γ rays)	Increases SIRT1 activity and testosterone levels in testis, and decreases oxidative stress response	El-Mesallamy et al. (2018)
Salvianic acid A	Phenolic acids	*Salvia miltiorrhiza Bunge*	L-02 cells (4 Gy ^60^Co γ rays)	Reduces apoptosis and DNA damage	Juan et al. (2012)
Salvianic acid A	Phenolic acids	*Salvia miltiorrhiza Bunge*	Balb/c mice (4, 8 Gy ^60^Co γ rays)	Protects hematopoietic system	Juan et al. (2015)
Resveratrol	Polyphenols	*Red grapes*	Female SD rats (3.2 Gy ^137^ Cs γ rays)	Inhibits NF-κB - activated inflammatory cytokines	Said et al. (2016)
Resveratrol	Polyphenols	*Red grapes*	Lymphocyte (0.5, 1 Gy X-rays)	Inhibits DNA damage	Basso et al. (2016)
Resveratrol	Polyphenols	*Red grapes*	Male C57BL/6 N mice (7 Gy ^137^ Cs γ rays)	Improves intestinal morphology, reduce crypt cell apoptosis, and regulates the expression of Sirt1 and p53	Zhang et al. (2017a)
Resveratrol	Polyphenols	*Red grapes*	Male C57BL/6 mice (6 Gy ^137^ Cs γ rays)	Ameliorates thymus and spleen atrophy, reduce lymphocyte count, Modulates immunosuppression	Zhang et al. (2018a)
Tea Polyphenols	Polyphenols	*Green tea*	Kunming mice (738 cGy ^60^Co γ rays)	Recovery the haematopoietic system, antioxidant potential activity and reduce inflammatory cytokines	Hu et al. (2011)
Tea Polyphenols	Polyphenols	*Green tea*	Male C57BL/6 mice (2 Gy X-rays)	Inhibits oxidative stress and mitochondrial apoptosis	Ding et al. (2015)
Ginseng oligopeptide	Oligopeptides	*Panax ginseng C.A.Mey.*	Caco-2 (2 Gy X-rays) Balb/c mice (3.5 Gy X-rays)	Attenuates immune dysfunction	He et al. (2017)
Ginseng oligopeptide	Oligopeptides	*Panax ginseng C.A.Mey.*	BALB/c mice (3.5, 8Gy ^60^Co γ rays)	Decreases inflammatory and oxidative stress	He et al. (2018)

### Saponins

Saponins are the main active compounds in many Chinese herbal medicines (Yang et al., 2014), especially in *Panax ginseng C.A.Mey*. Lee et al. established that the active ingredients in *Panax ginseng C.A.Mey* such as Ginsenoside Rc, Ginsenoside Rd, and Ginsenoside Re are radioprotective (Lee et al., 2006). The administration of Ginsenoside Rd and Ginsenoside Re in mice before irradiation enhanced the formation of endogenous splenic colonies and inhibited radiation-induced apoptosis of the intestinal crypt cells (Lee et al., 2006). Ginsenosides have a wide range of biological and pharmacological properties. They have been shown to be effective against neurological diseases, infectious diseases, and tumors (Rokot et al., 2016; Arring et al., 2018; Nguyen and Nguyen, 2019). Through intestinal biotransformation, *Panax ginseng C.A.Mey.* can be transformed into high pharmacological activity metabolites that act on multiple human tissues (Mancuso and Santangelo, 2017).

Other medicinal herbs have also been shown to contain saponins that play a role in radioprotection. Administration of Gypenoside before irradiation effectively increased serum superoxide dismutase (SOD) and CAT levels that inhibit the expression of Nrf2 and HO-1 (Ying et al., 2018). Saponins from *Eleutherococcus senticosus (Rupr. & Maxim.) Maxim.* ameliorate peripheral blood cell damage associated with radiation (Yue et al., 2005).

### Polysaccharides

Polysaccharides are widespread in animals, plants, and microorganisms. They from part of the primary substances that make up living things (Chen and Huang, 2018). *Panax ginseng C.A.Mey.* contains polysaccharides. The acidic polysaccharide of ginseng (APG) has been shown to increase IL-12 levels in bone marrow cells (BMs) of irradiated mice. Kim et al. speculated that APG contribute to the proliferation of CD4 (+) T lymphocytes and facilitate viability as well as alloreactivity by inducing phenotypic changes in BMs (Kim et al., 2007). APG inhibits the activation of the p53-dependent pathway and the mitochondrial apoptosis pathway. It down-regulates pro-apoptotic proteins (p53, BAX, cytochrome-c, and caspase-3), thereby, promoting the proliferation of crypt cells. These effects were shown to protect the small intestines of mice from radiation damage (Park et al., 2011; Bing et al., 2014).

*Lycium barbarum L.* is commonly used in traditional medicine to nourish the liver and kidney. Its active ingredient, the Lycium barbarum polysaccharide (LBP), exhibits significant antioxidant effects. Studies have reported that after multiple consecutive local ^60^Co γ-rays irradiation of rats’ testis, LBP enhanced testicular SOD activity, inhibited malondialdehyde (MDA) levels, promoted redox balance recovery, and restored the secretion of testosterone (Luo et al., 2011). In the hematopoietic system, LBP was shown to increase the antioxidant capacity of bone marrow mononuclear cells and mitigated cell cycle arrest by interfering with adhesion molecules CD44 and CD49d (Zhou et al., 2016). In addition, by acting on on Bcl-2 and Bax, LBP inhibits spermatogenic cell apoptosis by regulating mitochondrial membrane potential and inhibiting mitochondrial apoptosis (Luo et al., 2014).

*Astragalus mongholicus Bunge* is a qi-tonifying medicinal herb that is often used in qi deficiency syndromes (Li et al., 2014b). Radiation-induced lung injury is one of the most common and fatal complications of chest radiotherapy (Klein et al., 2016). After ionizing irradiation, alveolar epithelial cells in the lung exhibit a senescence phenotype and up-regulates the transcription of pro-inflammatory factors that induce pulmonary fibrosis (Beach et al., 2017). This condition manifests itself as breathlessness. The Astragalus polysaccharide is the active ingredient in *Astragalus mongholicus Bunge*. It inhibits thiobarbituric acid reactive substances and pro-inflammatory factors, but also activates SOD, catalase and glutathione. In addition, it reduces the damage of pulmonary fibrosis caused by peroxidation. Its mechanism of action is correlated with the expression of NF-κB, and this mechanism applys to radiation-induced liver injury (Liu et al., 2014).

Astragalus polysaccharide inhibits p38 phosphorylation, JNK, ERK1/2, NF-κB P65, and COX-2 protein expression levels. Evidence shows that it inhibits ionizing radiation-induced side effects through the ROS-mediated MAPK/NF-κB signaling pathway (Zhang et al., 2018c). Ionizing radiation decreases the capacity for cell proliferation. The Astragalus polysaccharide has been documented to promote self-renewal and proliferation of cells by elevating the expressions of peroxisome proliferator-activated receptor-γ (PPAR-γ), CCAAT/enhancer-binding protein α (C/EBPα) and protecting adipogenic differentiation functions (Kongxi et al., 2020). *In vitro* studies have also revealed that Astragalus polysaccharides enhance bone marrow mesenchymal stem cell proliferation by downregulating NF-κB signaling pathway-related proteins and maintaining DNA stability (Li-ying et al., 2018).

*Ganoderma Lucidum Karst* has qi replenishment and nerve soothing properties. It contains various biologically active components and pharmacological activities that are important in the control of multiple diseases (Jin et al., 2016; Ahmad, 2018). The Ganoderma lucidum polysaccharide is the main biologically active component of *Ganoderma Lucidum Karst*. Studies have shown that Ganoderma lucidum polysaccharide regulates the metabolism of endogenous substances (such as L-glutamic acid, taurine, and glycerophospholipid) that enhance the expression of relevant biomarkers in mice thymus and exert radioprotective effects (Kunmu et al., 2020). Guohui et al. reported that after exposing mice to radiation, *Ganoderma Lucidum Karst* increased mice survival rates and improved the phagocytic abilities of mononuclear macrophages and NK cells (Guohui et al., 2004).

The polysaccharides of *Angelica sinensis (Oliv.) Diels* have been documented to exhibit thymus and spleen protective indices, increase the number of red blood cells (RBC), white blood cells (WBC), and bone marrow cells of mice after irradiation. Therefore, they play a role in protecting the hematopoietic system (Zhao et al., 2012).

### Flavonoids

Flavonoids are widely distributed in plants and exhibit health promoting properties (Mukai, 2018). *Ginkgo biloba L.* contains flavonols and other active compounds (Mei et al., 2017) that are responsible for its free radical scavenging ability and antioxidant properties (Evans, 2013). Therefore, *Ginkgo biloba L.* prevents ionizing radiation mediated injuries by inhibiting oxidative stress. Sener et al. have detected rat lung, liver, kidney, and ileum. They reported that the administration of *Ginkgo biloba L.* before and after irradiation attenuated malondialdehyde (MDA) content and reduced DNA damage by inhibiting lipid peroxidation (Sener et al., 2006). *Houttuynia cordata Thunb.* has shown to clearing heat and removing toxicity, reducing swelling and draining pus (Ma et al., 2017). The flavonoid contents of *Houttuynia cordata Thunb.* have been shown to improve the state of peripheral blood cells after radiation, thereby reducing injury (Jun and Zhenghai, 2010).

### Glycosides

*Paeonia lactiflora Pall.* is an herb that nourishes the blood, restrains yin, softens the liver, and relieves pain. Paeoniflorin is a component of *Paeonia lactiflora Pall.*. Paeoniflorin increased glutathione (GSH), SOD, and reduced MDA and lactate dehydrogenase (LDH) content in an endothelial cell model. In addition, it was shown to reduce oxidative stress responses by interfering with the Nrf2/HO-1 pathway (Yu et al., 2013). Another study showed that it inhibited ROS-mediated mitochondrial apoptosis and reduced ROS accumulation as well as intracellular cytosolic Ca2 + concentrations. Other than inhibiting the mitochondrial apoptotic pathway by acting on Bcl-2, Bax, and caspase-3 (Li et al., 2007), paeoniflorin also inhibited MAPK signaling pathway activation and reversed radiation-induced DNA damage (Yang et al., 2015).

Astragaloside IV is an active ingredient in *Astragalus mongholicus Bunge* that is involved in controlling apoptosis or cell cycle, down-regulation of the Bax/Bcl-2 ratio, inhibition of G0/G1 cell-cycle arrest, increasing the proliferative ability of bone marrow cells, and in protection against radiation induced damage to the hematopoietic system (Li et al., 2011).

*Rhodiola rosea L.* contains a variety of biologically active compounds with antioxidant, anti-inflammatory, and stress response properties (Amsterdam and Panossian, 2016; Nabavi et al., 2016). Glycosides in *Rhodiola rosea L.* were shown to stimulate lymphocytic cell proliferation after radiation (Tianxiang et al., 2013).

### Phenolic Acids

*Angelica sinensis (Oliv.) Diels* promotes blood nourishment and circulation and is often used to treat blood deficiency syndromes. Ferulic acid (FA) is a bioactive component of *Angelica sinensis (Oliv.) Diels* that reduces DNA damage. Studies have shown that administration of FA to mice 1 h before or after irradiation inhibited micronuclei formation in peripheral blood. FA promotes hematopoietic recovery by attenuating DSBs in white blood cells and bone marrow cells (Maurya and Devasagayam, 2013). After DNA damage, the PARPI repair mechanism is activated and regulates inflammatory mediators (such as cytokines, chemokines, and inducible Nitric Oxide synthase) (Bai and Virag, 2012), while SIRT1 negatively regulates PARP1 (Caito et al., 2010). After the exposure of mice to ionizing radiation, it was found that PARP1 activities and intracellular calcium concentrations increased in the testis, while SIRT1 activities and expression significantly decreased. FA reversed the expression of SIRT1, maintained testosterone levels, and reduced oxidative stress, while regulating PARP1 and cytosolic calcium concentrations to ameliorate spermatogenesis disorders (El-Mesallamy et al., 2018). Accumulated ROS enhances p53 nuclear transport, expands ataxia capillaries, and activates mutant protein (ATM). Using a radiation damage mice model, Das et al. showed that FA enhanced the nuclear translocation of nuclear factor Nrf2 and activated the NHEJ repair pathway in response to ROS-mediated oxidative stress and DNA damage (Das et al., 2017a). In addition to inhibiting DNA damage and promoting DNA repair, FA was also involved in the regulation of inflammatory pathways and related factors. It was shown to inhibit the expression of Cox-2 and inducible nitric oxide synthase 2 (iNOS-2) after irradiation, control the phosphorylation/activation of IKKα/β and IκBα pathways, and regulate the downstream NF-κB nuclear translocation, thus ameliorating radiation-induced inflammation (Das et al., 2014).

In the intestines, FA was shown to interfere with the ROS/NF-κB/Nrf2/p53-caspase 3-PARP axis. In this processes, it enhanced the expression of Mn-SOD and Heme oxygenase-1 (HO-1) that inhibited peroxidation, regulated phosphatidylserine and mitochondrial membrane potential, and suppressed the activation of downstream mitochondrial apoptotic pathways (Das et al., 2017b). Therefore, FA can regulate cell cycle while inhibiting lipid peroxidation and radiation-induced cell apoptosis. Ionizing radiation activates stress marker cyclin (Cdc42) and down-regulates the activation of survival pathways by inhibiting the phosphorylation of phosphatidylinositol 3-kinase (PI3K) and serine/threonine kinase (Akt). The phosphatase gene (PTEN) is a critical molecule that regulates the survival pathway of PI3K/Akt. Ionizing radiation significantly increases the expression of PTEN that promotes cell cycle arrest and inhibits survival-related pathways. It has been demonstrated that FA lowers the overexpression of Cdc42, apoptotic proteins (p53, p21, Bax, and PTEN), and increases PI3K phosphorylation. Moreover, reduced cell cycle arrest inhibits lipid peroxidation while increasing SOD and catalase activities (Das et al., 2016). After radiation, the senescence phenotype of normal cells can be observed with the cells undergoing inflammation and fibrosis (Li et al., 2018b). Due to its antioxidant and anti-inflammatory aspects, FA has been shown to ameliorate these conditions.

*Salvia miltiorrhiza Bunge* is an alternative therapeutic option for cardiovascular and cerebrovascular diseases. It has anti-inflammatory, antioxidant, and anti-cancer biologic properties (Shi et al., 2019a). Salvianic acid A is the active compound in *Salvia miltiorrhiza Bunge* that has been shown to inhibit apoptosis and reduce ionizing radiation associated DNA damage (Juan et al., 2012). An *in vivo* assay established that salvianic acid A protects the hematopoietic system and improves survival after radiation (Juan et al., 2015).

### Polyphenols

Polyphenols exhibit antioxidant properties that inhibit DNA damage caused by peroxide free radicals (Fraga et al., 2019). Resveratrol is a vital plant antitoxin that possesses antioxidant and anti-inflammatory effects. Resveratrol inhibits NF-κB - activated inflammatory cytokine secretion by up-regulating the expression of peroxisome proliferation-activated receptor (PPAR-4) and SIRT1. These effects prevent ionizing radiation-induced premature ovarian failure (Said et al., 2016). *In vitro* experiments, Basso et al. demonstrated that resveratrol is involved in inhibiting DNA damage after radiation through the assessment of human lymphocyte DNA damage, repair kinetics, and histone deacetylase activity (Basso et al., 2016). It also improves the morphology of the small intestine, reduces crypt cell apoptosis, regulates Sirt1 and acetylated p53, and has a radioprotective role (Zhang et al., 2017a). Resveratrol improves thymic and spleen atrophy, lymphocyte counts, and proliferation caused by ionizing radiation. Moreover, it inhibits serum levels of IL-2, IL-4, IL-7, and IFN-γ thereby regulating immune functions (Zhang et al., 2018a). Green tea is also rich in polyphenols. Tea polyphenols can improve hematopoietic functions after irradiation (Hu et al., 2011), inhibit oxidative stress and mitochondrial apoptosis as well as prevent radiation induced spermatogenic cell death (Ding et al., 2015).

### Oligopeptides

The Ginseng oligopeptide (GOP) reduces the concentration of plasma diamine oxidase and LPS, and inhibits the secretion of IL-1 as well as TNF-α. It also protects the intestinal epithelial barrier by up-regulating the expression of tight junction proteins (ZO-1 and Occludin). It promotes intestinal repairs by suppressing the expression of apoptosis-related proteins (Bax and Caspase-3) and elevating lymphocyte (CD3 +, CD4 +, CD8 +) concentrations (He et al., 2017; He et al., 2018). He et al. reported that prophylactic administration of GOP exhibited radioprotective effects while post-treatment was beneficial for the quick repair of irradiation-induced injuries (He et al., 2017).

In summary, various radioprotective compounds occur in traditional Chinese medicines. This implies that the anti-radiation mechanisms of Chinese herbal medicines are multi-faceted. However, Chinese herbal contains sophisticated compounds. If only an active compound is used to explore the effect mechanism, there may be limitations.

## Chinese Herbal Medicines and the Anti-radiation Effects

Extracts from Chinese herbal medicines have been used to comprehensively elucidate on the anti-radiation mechanisms of Chinese Herbal Medicines (Table 2).

**TABLE 2:** Effects and Mechanisms of Chinese Herbal Medicines in ionizing radiation damage.

Names	Familia	Objects (model inducer,dose)	Pharmacological action/Mechanisms	References
*Panax ginseng C.A.Mey.*	Araliaceae	Outbred albino rats (4 Gy ^60^Co γ rays)	Inhibits carcinogenesis	Bespalov et al. (2014)
*Panax ginseng C.A.Mey.*	Araliaceae	Balb/c mice (8 Gy ^60^Co γ rays)	Protects the bone marrow and increases inflammatory factor	Song et al. (2003)
*Panax ginseng C.A.Mey.*	Araliaceae	Swiss albino mice (6 Gy ^60^Co γ rays)	Protects against radiation-induced hematological and biochemical alterations	Verma et al. (2011)
*Panax ginseng C.A.Mey.*	Araliaceae	Splenocytes (5 Gy ^60^Co γ rays) Balb/c mice (4.5 Gy ^60^Co γ rays)	Inhibits immunosuppression	Han et al. (2005)
*Panax ginseng C.A.Mey.*	Araliaceae	Wistar rats (6 Gy ^60^Co γ rays)	Protects against cardio-nephrotoxicity through enhancing the antioxidant activity and inhibition of endothelial dysfunction	Mansour (2013)
*Panax ginseng C.A.Mey.*	Araliaceae	C57BL/6 mice (15 Gy X-rays)	Prevents the manifestations of oxidative stress, protects against RILI	Jang et al. (2015)
*Panax ginseng C.A.Mey.*	Araliaceae	C57BL/6 mice(6.5 Gy ^60^Co γ rays)	Protects against radiation-induced inflammation and cancer	Koo et al. (2013)
*Panax ginseng C.A.Mey.*	Araliaceae	RAW264.7 cells (10 Gy ^60^Co γ rays)	Inhibits proinflammatory responses	Lee et al. (2014)
*Panax ginseng C.A.Mey.*	Araliaceae	RBL-2H3 cells (10, 30, 50, 70, 100 kGy ^60^Co γ rays) Balb/c mice (100 kGy ^60^Co γ rays)	Suppresses the release of β-hexosaminidase, histamine, intracellular ROS and Ca2 + influx induced by IgE-antigen complex, inhibits mast cell-mediated signal transduction activity, and reduces IL- 4 serum levels	Kang et al. (2018)
*Panax quinquefolius L.*	Araliaceae	Human (1, 2 Gy X-rays)	Inhibits lymphocytic DNA damage after radiotherapy, Increases total antioxidant capacity	Lee et al. (2010)
*Zingiber officinale roscoe*	Zingiberaceae	Swiss albino mice (6–12 Gy ^60^Co γ rays)	Inhibits lipid peroxidation	Jagetia et al. (2003)
*Zingiber officinale roscoe*	Zingiberaceae	Human mesenchymal stem cells (4 Gy ^60^Co γ rays)	Induces Nrf2 nuclear translocation	Ji et al. (2017)
*Zingiber officinale roscoe*	Zingiberaceae	SD rats (2 Gy ^60^Co γ rays)	Improves taste, vomiting symptoms	Sharma et al. (2005)
*Zingiber officinale roscoe*	Zingiberaceae	Albino rats (6 Gy ^60^Co γ rays)	Regulates inflammatory signaling pathways	Mohamed and Badawy (2019)
*Zingiber officinale roscoe*	Zingiberaceae	Albino rats (6 Gy ^60^Co γ rays)	Suppresses inflammatory factors, enhances mitochondrial complex activity	Soliman et al. (2018)
*Angelica sinensis (Oliv.) Diels*	Apiaceae	Wistar rats (8 Gy X-rays)	Inhibits OPN, C-Jun, miRna-21 expressions, reduces TGF- 1 release and Tn-I levels, resistance to radiation-induced cardiac fibrosis	Ma et al. (2019)
*Ginkgo biloba L.*	Ginkgoaceae	Wistar rats (6 Gy ^60^Co γ rays)	Inhibits lipid peroxidation and prevents DNA damage	Ismail and El-Sonbaty (2016)
*Ginkgo biloba L.*	Ginkgoaceae	Wistar rats(1 mci of (99 m)Tc)	Anti-lipid peroxidation and anti-apoptotic	Raafat et al. (2013)
*Portulaca oleracea L.*	Portulacaceae	Albino rats (6 Gy ^60^Co γ rays)	Relieves lipid peroxidation in liver and kidney	Abd El-Azime et al. (2014)
*Lycium barbarum L.*	Solanaceae	Kunming mice (5 Gy X-rays)	Inhibits the expression of P53, caspase-3 and caspase-6, and accelerates the recovery of splenic functions	Duan et al. (2015)
*Crataegus pinnatifida Bunge*	Rosaceae	Lymphocytes (150 cGy ^60^Co γ rays)	Reduces micronucleus in lymphocytes	Hosseinimehr et al. (2009)
*Hippophae rhamnoides L.*	Elaeagnaceae	Swiss albino strain ‘A’Male mice (10 Gy ^60^Co γ rays)	Protects mitochondria and chromatin	Shukla et al. (2006)
*Hippophae rhamnoides L.*	Elaeagnaceae	Swiss albino mice (5Gy, 10 Gy ^60^Co γ rays)	Enhances spermatogonial proliferation, stem cell survival and reduces sperm abnormalities	Goel et al. (2006)

### Roots and Rhizomes of Chinese Herbal Medicine

Studies have confirmed that *Panax ginseng C.A.Mey.* reduces the overall or local cancer rates induced by long-term exposures to radiation (Bespalov et al., 2014). Ionizing radiation causes acute myelosuppression and leads to the apoptosis of hematopoietic stem cells as well as hematopoietic progenitor cells. These pathological changes are the primary causes of death after exposure to moderate-to-high radiation doses (Shao et al., 2014). The extract of *Panax ginseng C.A.Mey.* was shown to increase bone marrow cells, spleen cells, and granulocyte-macrophage colony-forming units (CFU-GM) in mice while promoting the secretion of endogenous cytokines (IL-1, IL-6, and IL-12) to rejuvenate hematopoietic functions (Song et al., 2003). In addition, the extract inhibited the decrease in red blood cell counts, hemoglobin and hematocrit, as well as prevented radiation induced anemic symptoms (Verma et al., 2011). The extract of *Panax ginseng C.A.Mey.* has been reported to protect the hematopoietic system from ionizing radiation by inhibiting cyclooxygenase 2 (COX-2) and down-regulating activated p38 MAPK and PI3K/Akt pathways (Koo et al., 2013). Ionizing radiation-induced changes in the cellular microenvironment affect the immune system (Frey et al., 2017). *Panax ginseng C.A.Mey.* has immune regulation properties (Lee et al., 2005). Studies have shown that *Panax ginseng C.A.Mey.* elevates the mRNA expression of Th1 and Th2 cytokines and inhibits immunosuppression after radiation by stimulating normal spleen cells in mice (Han et al., 2005). *Panax ginseng C.A.Mey.* also exerts its radioprotective effects by inhibiting the expression of IL-1β in macrophages while simultaneously preventing the signal cascade of CHK2 and nuclear factor kappa B (NF-κB) (Lee et al., 2014). By destroying the intestinal epithelial barrier, radiation therapy enhances intestinal permeability and mucosal injury. Intestinal injuries lead to high plasma lipopolysaccharide (LPS) levels and elevated pro-inflammatory cytokine secretions that trigger a series of inflammatory reactions and bowel syndrome (Romesser et al., 2019). Moreover, *Panax ginseng C.A.Mey.* was shown to improve appetite in rats and reduced anorexic symptoms after radiation (Balaji Raghavendran et al., 2012). Ionizing radiation also causes damage to other tissues and organs. Oral administration of the extract of *Panax ginseng C.A.Mey.* before irradiation inhibited the suppression of serum creatine kinase and lactate dehydrogenase levels, suppressed urea and creatinine levels and further protected against irradiation-induced cardio-nephrotoxicity by enhancing antioxidant activities and inhibiting endothelial dysfunctions (Mansour, 2013). Through its antioxidant mechanisms, *Panax ginseng C.A.Mey.* can inhibit catalase activity, increase glutathione content, suppress the expression of IL-1β, TNF-α, and alleviates inflammation in radiation-induced lung injuries (Jang et al., 2015). Ionizing radiation leads acute skin damage (Park et al., 2018). The extract of *Panax ginseng C.A.Mey.* was shown to suppress the secretion of β-hexosaminidase, histamine, intracellular ROS, and internal Ca2+. It was also revealed that Black *Panax ginseng C.A.Mey.* inhibited mastocyte-mediated signaling activities, suppressed IL-4 serum levels, and ameliorated the symptoms and clinical signs of post-radiation allergic dermatitis (Kang et al., 2018). These studies suggest that *Panax ginseng C.A.Mey.* may be a useful herb against radiation associated damage.

*Panax quinquefolius L.* belongs to the Araliaceae family and has yin nourishment as well as heat clearing properties. From a clinical observation study, it was suggested that *Panax quinquefolius L.* ameliorated lymphocytic DNA damage after radiotherapy, suppressed micronucleus ratios in human lymphocytes, and enhanced total antioxidant capacities after exposure to radiation (Lee et al., 2010). In addition, this herb protects genes from acute damage in a short period. Studies have established that *Panax quinquefolius L.* tea protects cellular DNA from oxidative stress damage for at least 2 h (Szeto et al., 2015).

A fresh rhizome of *Zingiber officinale Roscoe* has been reported as being able to inhibit lipid peroxidation and excessive glutathione consumption (Jagetia et al., 2003), and improve mice survival after irradiation (Jagetia et al., 2004). Ji et al. demonstrated that the extract of *Zingiber officinale Roscoe* suppresses ionizing radiation-induced overproduction of ROS and DSBs in human mesenchymal stem cells. Its antioxidant mechanisms involve the induction of NRF2 nuclear translocation and activation of its downstream cell protection genes (HO- 1 and NQO-1) (Ji et al., 2017). Radiotherapy confers adverse side effects such as vomiting and nausea. These side effects have been attributed to ionizing radiation associated damage of the gastrointestinal, viscera and the vagus nerve to release serotonin that activates the brains vomiting center through serotonin receptors. Furthermore, radiation affects neural activity in the brain and activates specific sensory receptors. *Zingiber officinale Roscoe* can ameliorate nausea and vomiting. *Zingiber officinale Roscoe* inhibits the activation of related receptors, promotes neurobehavioral functions, and alleviates radiation-induced taste aversion and vomiting (Sharma et al., 2005). Besides, *Zingiber officinale Roscoe* plays a role in the regulation of inflammatory signaling pathways. Zingerone suppresses the MAPK signaling pathway, inhibits cytochrome P4502E1 as well as nicotinamide adenine dinucleotide phosphate (NADPH) oxidase, and downregulates liver enzyme activities. Further, it has been shown to regulate the expression of inflammatory markers such as TLR4, iNOS, COX-2, and MPO (Mohamed and Badawy, 2019). Negative regulation of the TLR4 pathway by zingerone alleviates radiation-induced hepatic injury (Lee et al., 2018). Epidemiological studies have indicated that adults with congenital heart diseases are exposed to low-dose ionizing radiations during cardiac surgeries. These patients have an increased risk for cancer when compared to the general population (Cohen et al., 2018). Studies have shown that prophylactic administration of zingerone regulates serum lactate dehydrogenase, creatine kinase-MB activity and suppresses the expression of TNF-α as well as COX-2. It also inhibits DNA fragmentation, enhances mitochondrial complex activity, and interferes with the aggravation of ionizing radiation induced heart damage (Soliman et al., 2018). The antioxidant and anti-inflammatory effects of *Zingiber officinale Roscoe* in other tissue damage models have not been established.

Chest radiotherapy induces myocardial fibrosis (Curigliano et al., 2016) while whole-body radiation can lead to osteopontin (OPN) activation. OPN is a cytokine involved in myocardial fibrosis. The extract of *Angelica sinensis (Oliv.) Diels* inhibits radiation induced cardiac fibrosis by suppressing the expression of OPN, c-jun, and miRNA-21 as well as suppressing Troponin-I (Tn-I) levels (Ma et al., 2019). The main active components in *Paeonia lactiflora Pall.* have been shown to have radioprotective properties.

### Leafy Chinese Herbal Medicines

Different parts of *Ginkgo biloba L.* in the Ginkgo family can be used as alternative medicine. *Ginkgo biloba L. leaves* have been utilized in studies of radiation-associated injuries. Ionizing radiation induces permanent nervus cerebral defects, chronic microangiopathy, and blood-brain barrier dysfunctions. Radiation associated brain damage results in cerebrovascular abnormalities, demyelination, white matter necrosis, and cognitive impairment (Lumniczky et al., 2017). Abnormally elevated levels of catecholamine, epinephrine, norepinephrine, dopamine, and inflammatory factors are the major causes of radiation-induced brain injuries. Ismail et al. confirmed that the extract of *Ginkgo biloba L.* regulated the above mentioned indicators by suppressing lipid peroxidation (Ismail and El-Sonbaty, 2016). In addition, that the extract of *Ginkgo biloba L.* was shown to suppress the expressions of P53, Bcl-2 and inhibited apoptosis after radiation (Raafat et al., 2013).

### Whole Chinese Herbal Medicines

Biologically active compounds in *Portulaca oleracea L.* include flavonoids, alkaloids, terpenoids, and sterols. These compounds have been shown to have antioxidant, anti-bacterial, and anti-inflammatory properties. *Portulaca oleracea L.* extracts alleviated lipid peroxidation in the liver and kidney of irradiated rats. It also suppressed MDA levels in tissues and inhibited total cholesterol (TC), triglyceride (TG), low-density lipoprotein cholesterol (LDL-c), and maintained atherosclerosis indices (Abd El-Azime et al., 2014). From the mechanism perspective, *Portulaca oleracea L.* may also play an anti-inflammatory role by inhibiting TNF-α secretion, thereby preventing NF-κB nuclear translocation. This herb also plays an important role in regulating peroxidation (Zhou et al., 2015). *Mentha canadensis L.* from the Labiatae family inhibits radiation induced damage by scavenging for free radicals, its antioxidant, anti-inflammatory, anti-mutation activities, and enhancing DNA repair (Baliga and Rao, 2010).

### Fructus of Chinese Herbal Medicines

The antioxidant properties of the Solanaceae *Lycium barbarum L.* are important in antagonizing mitochondrial apoptosis and inhibiting DNA damage. Duan et al. documented that *Lycium barbarum L.* increases the DNA content of red blood cells and hemoglobin, effectively inhibits P53, caspase-3, and caspase-6 while accelerating the recovery of splenic functions (Duan et al., 2015). *Crataegus pinnatifida Bunge* is rich in polyphenols and total flavonoids. An extract of *Crataegus pinnatifida Bunge* has been established to reduce lymphocytic micronucleus and lowers the effects of radiation (Hosseinimehr et al., 2009). While an extract of *Hippophae rhamnoides L.* scavenges for free radicals, prevents cell cycle arrest in the G2-M phase (Goel et al., 2003), and protects mitochondria and chromatin from radiation-induced damage (Shukla et al., 2006). Moreover, *Hippophae rhamnoides L.* protects against radiation-induced sperm injuries (Goel et al., 2006).

In conclusion, the above reviews show that a single Chinese herbal medicine can ameliorate radiation-induced damage in a variety of ways. These herbs have considerable potential as radioprotectants.

### Chinese Herbal Prescriptions in the Prevention of Radiation Damage

Traditional Chinese Medicine’s clinical efficacy exerted in the form of Chinese herbal prescriptions, is based on clinical symptoms and purposefully matched different Chinese medicines. Experimental and clinical studies have verified the therapeutic effect of Chinese Herbal Prescriptions (Zhang et al., 2016; Gao et al., 2017). Compared to a single herb or active compound, Chinese Herbal Prescription contains many active compounds with multiple therapeutic targets. These Prescriptions are suitable for the prevention and treatment of multiple ionizing radiation induced systemic damage. Synergism between the different herbs enhance therapeutic effects with reduced toxicity (Zhang et al., 2018b) (Table 3).

**TABLE 3:** Effects and Mechanisms of Chinese Herbal Prescription in ionizing radiation damage.

Chinese Herbal Prescription	Content	Objects (model inducer,dose)	Pharmacological action/Mechanisms	References
Siwu Tang	*Rehmannia glutinosa (Gaertn.) DC., Angelica sinensis (Oliv.) Diels, Conioselinum anthriscoides 'Chuanxiong', Paeonia lactiflora Pall.*	Female C57BL/6 mice (3.5 Gy ^60^Co γ rays))	Promotes hematopoietic and immune system recovery	Liang et al. (2006) and Liu et al. (2017)
BuzhongYiqi Tang	*Astragalus mongholicus Bunge, Codonopsis pilosula (Franch.) Nannf., Atractylodes lancea (Thunb.) DC., Bupleurum chinense DC., Actaea cimicifuga L., CCitrus reticulata Blanco, Angelica sinensis (Oliv.) Diels, Glycyrrhiza glabra L.*	ICR mice (3 Gy ^60^Co γ rays)	Increases the peripheral white blood cell count, relieves platelet damage, reduces lipid peroxides and improves the hematopoietic microenvironment	Xiao-fang et al. (2013) and Xiaoling et al. (2006)
Xuebijing (XBJ) injection	*Carthamus tinctorius L., Paeonia lactiflora Pall., Conioselinum anthriscoides 'Chuanxiong', Salvia miltiorrhiza Bunge, and Angelica sinensis (Oliv.) Diels*	ICR mice (2 Gy, 7.5Gy ^60^Co γ rays) Bone marrow mononucleated cells (1 Gy, 4Gy ^60^Co γ rays)	Reduces ROS in bone marrow cells	Li et al. (2014a)
Yiqi Yangyin Fang (YYF)	*Astragalus mongholicus Bunge, Panax ginseng C.A.Mey., Ligustrum lucidum W.T.Aiton, Eclipta prostrata (L.) L., Angelica sinensis (Oliv.) Diels, Atractylodes macrocephala Koidz., Poria cocos (Schw.) Wolf, Glycyrrhiza glabra L.*	ICR mice (2 Gy, 4Gy ^137^ Cs γ rays)	Increases the number of bone marrow cells, hematopoietic progenitor cells, and hematopoietic stem cells, inhibits bone marrow suppression by reducing intracellular ROS levels	Zhang et al. (2017b)
HemoHIM	*Angelica sinensis (Oliv.) Diels, Ligusticum officinale (Makino) Kitag., Paeonia lactiflora Pall.*	Female C57BL/6 mice (5 Gy ^137^ Cs γ rays)	Regulates IL-12 p70/pSTAT4 signaling pathway, accelerates the recovery of immune cells	Park et al. (2012) Park et al. (2014)
Bushen Jiedu Recipe	*Schisandra chinensis (Turcz.) Baill., Ophiopogon japonicus (Thunb.) Ker Gawl., Codonopsis pilosula (Franch.) Nannf., Astragalus mongholicus Bunge*	Knockout mice (6 Gy ^60^Co γ rays)	Regulates TLR4 signaling pathway, reduces white blood cell damage, and protects immune organs	Yunjing et al. (2015) and Lidan et al. (2016)
Wumai Danghuang Oral Liquid	*Schisandra chinensis (Turcz.) Baill., Ophiopogon japonicus (Thunb.) Ker Gawl., Codonopsis pilosula (Franch.) Nannf., Astragalus mongholicus Bunge*	Kunming mice (2 Gy, 3 Gy ^60^Co γ rays)	Increases SOD activity, degrades MDA content, repairs the immune system	Chunhong et al. (2014) and Hang et al. (2013)
Co-Herba Houttuyniae Oral Liquid	*Houttuynia cordata Thunb., Panax ginseng C.A.Mey., Lycium barbarum L.*	Kunming mice (1.5 Gy, 3 Gy ^60^Co γ rays)	Reduces the rate of chromosomal aberrations, enhances immune functions and the anti-stress ability	Lin et al. (2001) and Lin et al. (2002)
Radioprotection Formula	*Astragalus mongholicus Bunge,Ganoderma Lucidum Karst, Lycium barbarum L., Poria cocos (Schw.)Wolf*	Kunming mice (3 Gy, 7.5 Gy ^60^Co γ rays)	Increases the survival rate, white blood cell count, thymus index, spleen index and bone marrow cell DNA content	Liming et al. (2011)
STW 5	*Iberis amara L., Melissa officinalis L., Matricaria chamomilla L., Carum carvi L., Mentha aquatica L., Angelica archangelica L., Silybum marianum (L.) Gaertn., Chelidonium majus L., Glycyrrhiza glabra L.*	Male Wistar rats (6 Gy ^60^Co γ rays)	Inhibita oxidative stress responses, lowers inflammatory factors and intestinal damage index, regulates apoptosis-related factors	Khayyal et al. (2014) and El-Ghazaly et al. (2015)
Astragalus immortal prescription	*Rehmannia glutinosa (Gaertn.) DC., Ophiopogon japonicus (Thunb.) Ker Gawl. and Equus asinus L., Astragalus mongholicus Bunge*	Kunming mice (8 Gy X rays)	Increases the activities of GSH-Px, SOD and reduces MDA content in the liver	Cai-qin et al. (2017)
Wuzi Yanzong Pill (WZYZ)	*Lycium barbarum L., Cuscuta chinensis Lam., Schisandra chinensis (Turcz.) Baill., Plantago asiatica L., Rubus chingii Hu*	Male Kunming mice (4 Gy X rays)	Increases serum testosterone and reduces MDA and oxidative stress index (OSI)	Ji et al. (2016)
Yiqi Jiedu Decoction (YQJD)	*Astragalus mongholicus Bunge, Angelica sinensis (Oliv.) Diels, Lycium barbarum L., Panax quinquefolius L., Paeonia lactiflora Pall., Crataegus pinnatifida Bunge, Poria cocos(Schw.)Wolf, Portulaca oleracea L.*	Male Balb/c mice (2 Gy ^60^Co γ rays)	Promotes testicular index and testicular structure recovery, decreases spermatogenic cell apoptosis, and protects spermatogenic functions by intervening in TLR5 signaling pathways	Wang et al. (2020)

### Hematopoietic System

The hematopoietic system is highly sensitive to radiation. Hematopoietic cells exposed to ionizing radiation degenerate quickly, and undergo necrosis as well as apoptosis. In addition, ionizing radiation decreases the number of peripheral blood cells, especially neutrophils, lymphocytes, and platelets, leading to bleeding and anemia.

Siwu Tang is a classical Chinese herbal prescription that reinforces qi and nourishes the blood. It is comprised of four Chinese herbal medicines (*Rehmannia glutinosa (Gaertn.) DC.*, *Angelica sinensis (Oliv.) Diels*, *Conioselinum anthriscoides ‘Chuanxiong'*, and *Paeonia lactiflora Pall.*) (Sun et al., 2016). The active compounds in this prescription include fructose, paeoniflorin, and ferulic acid, among others. This Prescription promotes hematopoietic and immune system recovery after irradiation by increasing the number of peripheral white blood cells and bone marrow colony-forming units (Liang et al., 2006). Studies revealed that Siwu Tang induces the antioxidant Nrf2 pathway, partially inhibits DNA damage, prevents the activation of nuclear transcription factor activating protein-1 (AP-1) and NF-κB, thereby, inhibiting ionizing radiation-induced damage and oncogenesis (Liu et al., 2017).

Buzhong Yiqi Tang is a well-known Chinese herbal prescription with nearly 800 years of application. It is widely used a therapeutic option for spleen-qi deficiencies and post-illness symptoms (Hu et al., 2019; Liu et al., 2019). This prescription is composed of *Astragalus mongholicus Bunge*, *Codonopsis pilosula (Franch.) Nannf.*, *Atractylodes lancea (Thunb.) DC.*, *Bupleurum chinense DC.*, *Actaea cimicifuga L.*, *Citrus reticulata Blanco*, *Angelica sinensis (Oliv.) Diels*, and *Glycyrrhiza glabra L.*. This prescription has been shown to significantly elevate peripheral white blood cell counts in mice after irradiation and relieves platelet damage (Xiao-fang et al., 2013). Further, it suppresses lipid peroxides generated by the accumulation of free radicals and improves the hematopoietic microenvironment (Xiaoling et al., 2006).

Xuebijing (XBJ) is an injection of Chinese herbal prescription. It was approved by the National Medical Products Administration for the clinical management of septicemia. It contains *Carthamus tinctorius L.*, *Paeonia lactiflora Pall.*, *Conioselinum anthriscoides 'Chuanxiong'*, *Salvia miltiorrhiza Bunge*, and *Angelica sinensis (Oliv.) Diels*. A previous study documented that XBJ improved the survival rate of irradiated mice by suppressing ROS production in bone marrow cells and alleviated radiation induced hematopoietic cell injury (Li et al., 2014a).

In the study of Yiqi Yangyin Fang (YYF: *Astragalus mongholicus Bunge*, *Panax ginseng C.A.Mey.*, *Ligustrum lucidum W.T.Aiton*, *Eclipta prostrata (L.) L.*, *Angelica sinensis (Oliv.) Diels*, *Atractylodes macrocephala Koidz.*, *Poria cocos (Schw.) Wolf*, *Glycyrrhiza glabra L.*), Junling Zhang et al. indicated that this prescription increased the number of bone marrow cells, hematopoietic progenitor cells, and hematopoietic stem cells. It also improved bone marrow suppression after radiation by suppressing intracellular ROS levels (Zhang et al., 2017b).

### Immune System

Ionizing radiation damages the hematopoietic system, the thymus and the spleen. Radiation inhibits tissue and cell repair after injury and induces immunosuppression. Inhibition is proportional to the radiation dose. Hematopoietic and immune dysfunctions associated with ionizing radiation elevate tissue permeability, weaken body resistance, and predisposes the body to endogenous or exogenous infections.

HemoHIM is a prescription composed of three herbs with various biological and immunological activities. HemoHIM inhibits the continuous down-regulation of Th1 immune responses after radiation by regulating the IL-12 p70/pSTAT4 signaling pathway (Park et al., 2012). This prescription has also been shown to protect hematopoietic stem cells and speeds up the recovery of immune cells (Park et al., 2014).

Bushen Jiedu Recipe is an optimized combination of Liuwei Dihuang Pills. This prescription maintains kidney tone, nourishes yin, tonifies Qi and blood, clears heat and removes toxin. Yunjing et al. documented that Bushen Jiedu Recipe interfered with the expression of NF-κBp65 by regulating the TLR4 signaling pathway, suppressed white blood cell damage and protected the thymus and spleen (Yunjing et al., 2015; Lidan et al., 2016).

The Wumai Danghuang Oral Liquid is composed of *Schisandra chinensis (Turcz.) Baill.*, *Ophiopogon japonicus (Thunb.) Ker Gawl.*, *Codonopsis pilosula (Franch.) Nannf.*, and *Astragalus mongholicus Bunge.* This prescription exhibits its anti-radiation effects by elevating SOD, degrading MDA and inhibiting the generation of free radicals (Chunhong et al., 2014). In addition, Wumai Danghuang Oral Liquid exhibits protective and repair effect on radiation induced immune injuries (Hang et al., 2013).

*Houttuynia cordata Thunb.* and its bioactive molecules have anti-inflammatory and antioxidant properties (Shingnaisui et al., 2018). Co-Herba Houttuyniae Oral Liquid contains *Houttuynia cordata Thunb.*, *Panax ginseng C.A.Mey.*, *Lycium barbarum L.*. This liquid suppresses the rate of radiation induced chromosomal aberrations (Lin et al., 2001). It also enhances the anti-stress ability of mice by improving immune functions. Moreover, *Panax ginseng C.A.Mey.* and *Lycium barbarum L.* improve immune functions and inhibit radiation effects by promoting the repair of damaged cells and tissues (Lin et al., 2002).

The radioprotective prescription (*Astragalus mongholicus Bunge*, *Ganoderma Lucidum Karst*, *Lycium barbarum L.,* and *Poria cocos (Schw.)Wolf*) has been shown to improve the survival rate, white blood cell count, thymus index, spleen index, and the DNA content of bone marrow cells thereby reducing radiation induced immune damage in mice models (Liming et al., 2011).

### Digestive System

Ionizing radiation severely damages the digestive system and causes pathological changes such as intestinal mucosal damage and liver fibrosis. These damages lead to digestive and absorptive dysfunctions, resulting in a series of clinical symptoms like diarrhea, nausea, and vomiting.

STW 5 is a herbal prescription with anti-inflammatory and antioxidant properties. This prescription inhibits oxidative stress responses, suppresses the levels of inflammation factors, and intestinal damage indices by regulating apoptosis-related factors to prevent intestinal mucosal damage after radiation (Khayyal et al., 2014). Prophylactic administration of STW 5 reduces the severity of radiation mucositis (El-Ghazaly et al., 2015).

Astragalus immortal prescription is composed of *Rehmannia glutinosa (Gaertn.) DC.*, *Ophiopogon japonicus (Thunb.) Ker Gawl.* and *Equus asinus L.*. It is derived from Dunhuang medical papers (now stored in France, code: P.4038), and *Astragalus mongholicus Bunge*. This prescription has been shown to elevate the activities of GSH-Px and SOD while suppressing MDA levels in the liver. Furthermore, it protects the liver from radiation induced oxidative damage (Cai-qin et al., 2017).

### Reproductive System

The male reproductive system is very sensitive to ionizing radiation as it can damage the seminiferous epithelium and spermatogenic cells at all levels. These radiations can confer injuries to the reproductive system, and cause male infertility (Bates et al., 2016; Kesari et al., 2018). Radiation also causes DNA damage in spermatogenic cells, increases embryonic mortality, and offspring cancer susceptibility. It may induce hereditary changes.

Wuzi Yanzong Pill (WZYZ) is a Chinese herbal prescription that is used as a therapeutic option for male infertility. Clinically, it has significant therapeutic effects on oligospermia and asthenozoospermia. WZYZ improves sperm quality by suppressing DNA damage (Zhao et al., 2018). A double-blind randomized controlled trial confirmed that WZYZ is an excellent therapeutic option for men with low fertility who cannot be cured by conventional western medicines (Zhao et al., 2019). Pelvic exposure to radiation reduces testicular weight, sperm quality and leads to testicular oxidative stress and abnormal testicular structure. WZYZ protects against suppressed serum testosterone levels, reduces MDA levels and oxidative stress indices (OSI) in the testis. Its mechanism may be associated with up-regulation of PCNA (Ji et al., 2016).

The Yiqi Jiedu Decoction (YQJD) enhances testicular index, structural recovery of the testis, decreases the apoptotic rate of spermatogenic cells, and maintains spermatogenic functions after irradiation. These results suggest that YQJD plays a protective role by intervening in TLR5 downstream signaling pathways (Wang et al., 2020).

## Anti-Radiation Mechanisms of Chinese Herbal Medicines

### Mitochondrial Apoptosis Pathway

Oxidative stress leads to the pathogenesis of various human diseases and the aging process. Mitochondria is the energy center in cell metabolism, it regulates redox homeostasis, and plays a central role in diseases pathogenesis (Li et al., 2019a). Oxidative stress damages the mitochondria, accelerates excessive ROS production, activates the mitochondrial apoptotic pathway and induces apoptosis (Kim and Kim, 2018). Ionizing radiation mediated overproduction of ROS is associated with mitochondrial dysfunctions. ROS acts as a signaling molecule that initiates a series of cascade reactions (Wu et al., 2019). Changes in the mitochondrial membrane potential elevates the expressions of pro-apoptotic proteins and suppresses the expression of anti-apoptotic proteins (Sun et al., 2018). These events trigger the activation of caspase-3, and initiates the mitochondrial-dependent pathway (Li et al., 2018a). As previously stated, Chinese Herbal Medicines inhibit mitochondrial apoptosis by: i. inhibiting oxidative stress responses and suppressing the generation of ROS, and ii. Interfering with the expression of pathway associated factors, regulating the pro-apoptotic protein and anti-apoptotic protein ratios, as well as by suppressing Cytochrome C and caspase-3.

### MAPK Signaling Pathway and PI3K/AKT Signaling Pathway

Mitogen-activated protein kinases (MAPK) family, including three significant members of extracellular signal-regulated kinases (ERK), p38 kinase, and c-Jun N-terminal kinase (JNK), participate in various physiological processes such as morphogenesis, cell growth, proliferation, apoptosis, and differentiation (Fang and Richardson, 2005; Lu et al., 2019). Ionizing radiation activates the classic MAPK signaling pathway, JNK, and P38 MAPK pathways. In addition, radiation-induced secreted cytokines enhance MAPK pathway responses in cells (Dent et al., 2003). Enhancement of the MAPK signal upregulates telomerase activity, initiates changes in chromatin distribution, and regulates the cell cycle (Shain et al., 2018). Activated p38 and JNK signaling pathways are involved in immune regulation (Wang et al., 2019a). After p38 activation, a mitochondrial apoptotic pathway is initiated (Choi et al., 2006; Niaudet et al., 2017). The PI3K/AKT signaling pathway is essential in the regulation of cell growth, migration, proliferation, and metabolism in mammals (Pompura and Dominguez-Villar, 2018). Activated PI3K/Akt pathway accelerates DSB repair. Ionizing radiation inhibits its activation (Toulany and Rodemann, 2015). This pathway is also involved in cell cycle and apoptosis regulation after exposure to ionizing radiation (Chen et al., 2018). Because of the wide reach of the MAPK and PI3K/AKT signaling pathways, Chinese Herbal Medicines regulate them through multiple targets.

### Nrf2/HO1 Signaling Pathway

The Nrf2/HO-1 signaling pathway antagonizes tissue and organ oxidative stress injuries by regulating antioxidant, anti-inflammatory, apoptosis, pyroptosis, ferroptosis, and autophagy processes. Nrf2 is a transcription factor and the core regulator of cellular redox. It stimulates gene expression through antioxidant response elements in gene promoters and protects cells against ROS induced DNA damage (Zimta et al., 2019). When exposure to ionizing radiation occurs, Nrf2 acts as a critical transcription factor that regulates antioxidant enzymes and protects tissues from oxidative stress damage. HO-1 regulates the expression of apoptotic and inflammatory factors. In addition, it also promotes angiogenesis by preventing oxidative damage (Loboda et al., 2016). Chinese Herbal Medicines trigger Nrf2 and enhances mRNA and protein expressions of HO-1. These events trigger the antioxidant pathway and inhibits ionizing radiation induced oxidative damage.

### Inflammatory Signaling Pathway

Ionizing radiation-induced DNA damage results in phosphorylation and activation of multiple transcription factors (such as NF-κB, p53, and MAPK) by stimulated ATM kinases. ROS is also involved in these processes (Purbey et al., 2017). After exposure to ionizing radiation, the secretion of various inflammatory cytokines (IL-1, IL-6, TNF-α, IFN-γ, COX-2) is elevated. Inflammatory cytokines recruit immune cells that regulate cell microenvironment with a crucial impact on local or systemic tissues (Harding et al., 2017). Chinese Herbal Medicines exhibit a two-way regulation effect on inflammatory factors. The first one is by inhibiting the secretion of inflammatory factors, preventing fibrosis and inflammatory lesions after irradiation. The second strategy is that it plays an immunomodulatory role by regulating signaling pathways such as TLRs and NF-κB to reduce apoptosis (Scholch et al., 2015; Liu et al., 2018).

**FIGURE 2:**
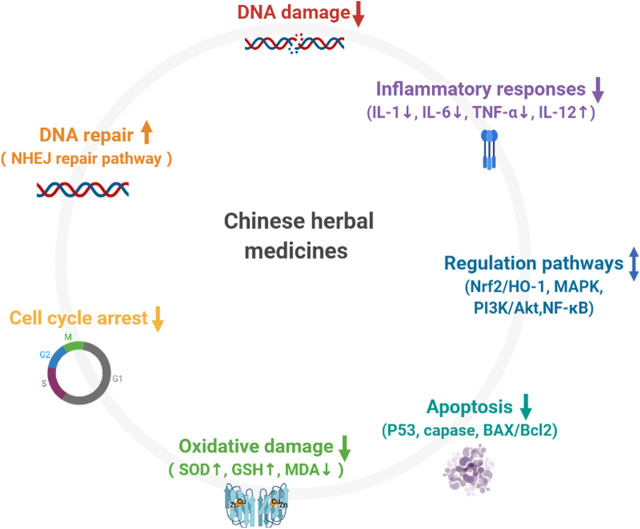
Mechanism of Chinese herbal medicines in preventing radiation injury. 1) Chinese herbal medicines reduce DNA damage; 2) Chinese herbal medicine promote DNA repair; 3) Chinese herbal medicines regulate cell cycle arrest; 4) Chinese herbal medicines prevent excessive accumulation of ROS and inhibits oxidative damage; 5) Chinese herbal medicine suppress extrinsic and intrinsic apoptotic pathways via reduction of P53, caspase, BAX/Bcl2 activation; 6) Chinese herbal medicines are involved in the regulation of inflammatory response; 7) Chinese herbal medicines participate in the regulation of multiple abnormally activated signaling pathways.

Traditional Chinese Medicine exhibits its curative effects on multiple body systems (such as hematopoiesis, immunity, reproduction, respiration, and circulation) by inhibiting oxidative stress, reducing DNA damage, and regulating abnormally activated signaling pathways. The Mechanism is as shown in Figure 2. It is worth noting that Chinese Herbal Medicines are particularly useful in anti-lipid peroxidation. Recently, a new regulatory cell death method (ferroptosis) has attracted considerable attention. Excessive ROS results in membrane lipid peroxidation. The accumulation of iron-dependent lipid peroxides leads to ferroptosis. Radiation induced damage to the hematological system and the lungs can be relieved by intervening in ferroptosis-related pathways (Li et al., 2019c; Zhang et al., 2020). This is an avenue for Traditional Chinese Medicine research in future.

## Conclusion

Ionizing radiation injuries are systemic damages that affect multiple organs and tissues. The Traditional Chinese Medicine characteristic theory lies in its holistic view: man and nature as a harmonious and unified whole, emphasizing the interactions between man and the environment, achieving a balance between the two. Simultaneously, various systems of the human body as a whole are connected physiologically and pathologically influence each other. Based on this holistic view, Chinese medicines and Chinese Herbal Prescriptions are suitable for use as therapeutic options for multi-system damages caused by ionizing radiation.

Compared to a single herb, the composition of Chinese Herbal Prescription is more complex and exhibits its therapeutic effects by having multiple targets (Yang et al., 2017; Wang et al., 2019b). The synergy between the Chinese medicines in the Chinese Herbal Prescriptions improves its efficacy while inhibiting toxic and side effects. A rationally designed Chinese Herbal Prescription will exhibit a better protective effect.

Chinese medicine has favorable economic benefits and is an economical option for the development of safe and effective radioprotectors. With positive effects, many Chinese Herbal Prescriptions have been used in clinical settings to reduce radiation induced damage. Studies on the anti-radiation activities and mechanisms of single Chinese medicines and their active compounds are limited. However, due to their sophisticated active compounds, it is difficult to elucidate on their potential radio-protective mechanisms. More studies are needed to evaluate the efficacy of these medicines. Systemic biology and network pharmacology applications may provide alternative methods and strategies for the applications of Chinese Herbal Prescriptions (Boezio et al., 2017; Tavassoly et al., 2018), and may, therefore, help in the development of innovative drugs for radiation protection.
